# Two types of anaesthesia and length of hospital stay in patients undergoing unilateral total knee arthroplasty (TKA): a secondary analysis based on a single-centre retrospective cohort study in Singapore

**DOI:** 10.1186/s12871-021-01459-7

**Published:** 2021-10-11

**Authors:** Xuan JI, Weiqi KE

**Affiliations:** 1 Department of Traditional Chinese Medicine, The First Affiliated Hospital of Shantou University Medical College, Shantou, Guangdong Province China; 2 Department of Anesthesiology, The First Affiliated Hospital of Shantou University Medical College, 57 Changping Road, Shantou, 515041 Guangdong Province China

**Keywords:** Total knee arthroplasty, General anaesthesia, Regional anaesthesia, Length of stay

## Abstract

**Background:**

Evidence regarding the relationship between the type of anaesthesia and length of hospital stay is controversial. Therefore, the objective of this research was to investigate whether the type of anaesthesia was independently related to the length of hospital stay in patients undergoing unilateral total knee arthroplasty (TKA) after adjusting for other covariates.

**Methods:**

The present study was a cohort study. A total of 2622 participants underwent total knee arthroplasty (TKA) at a hospital in Singapore from 2013 to 1-1 to 2014-6-30. The target independent variable and the dependent variable were two types of anaesthesia and length of hospital stay, respectively. The covariates included age, BMI, hemoglobin (Hb), length of stay (LOS), duration of surgery, sex, ethnicity, American Society of Anesthesiologist (ASA) Status, smoking, obstructive sleep apnea (OSA), diabetes mellitus (DM), DM on insulin, ischemic heart disease (IHD), congestive cardiac failure (CCF), cerebrovascular accident (CVA), creatinine > 2 mg/dl, day of week of operation. Multivariate linear and logistic regression analyses were performed on the variables that might influence the choice of the two types of anaesthesia and the LOS. This association was then tested by subgroup analysis using hierarchical variables.

**Results:**

The average age of 2366 selected participants was 66.57 ± 8.23 years old, and approximately 24.18% of them were male. The average LOS of all enrolled patients was 5.37 ± 4.87 days, 5.92 ± 6.20 days for patients receiving general anaesthesia (GA) and 5.09 ± 3.98 days for patients receiving regional anaesthesia (RA), *P* < 0.05. The results of fully adjusted linear regression showed that GA lasted 0.93 days longer than RA (β = 0.93, 95% CI (0.54, 1.32)), *P* < 0.05. The results of fully adjusted logistic regression showed that LOS > 6 days was 45% higher for GA than for RA (OR = 1.45, 95% CI (1.15, 1.84)), *P* < 0.05. Through the subgroup analysis, the results were basically stable and reliable.

**Conclusion:**

Our study showed that GA increased the length of stay during unilateral TKA compared with RA. This finding needs to be validated in future studies.

## Introduction

Total knee arthroplasty (TKA) is a radical surgery for the treatment of pain, movement limitation and joint deformity caused by osteoarthritis, rheumatoid arthritis and knee joint trauma. With the continuous development of TKA and the application of the concept of accelerated rehabilitation surgery in TKA, patients who undergo TKA now have less trauma, less bleeding, faster recovery and a significantly shorter length of stay (LOS), and some patients can even be discharged the day of surgery. Shortening the LOS can increase the turnover of inpatients, reduce the waste of medical resources, reduce medical expenses and lighten the social burden of medical treatment [1]. The LOS after TKA is closely related to prognosis; however, there are many factors that affect LOS [2], and there have been some studies linking the type of anaesthesia to the LOS [3]. At present, the two most commonly used types of anaesthesia by broad category are general anaesthesia (GA) and regional anaesthesia (RA). In this study, GA includes endotracheal intubation anaesthesia and RA includes spinal anaesthesia and epidural anaesthesia. Previous research has established that both types of anaesthesia can be used in TKA. However, findings from previous studies regarding the relationship between type of anaesthesia and LOS are controversial. In some studies, no association between the type of anaesthesia and LOS was found in multivariable analyses [4]. In contrast, some other studies suggested that the type of anaesthesia was related to the LOS [5, 6]. Given the differences in research design, target population, and data analysis of these studies, the performance of additional studies remains important. Previous studies have shown that different types of anaesthesia can increase or decrease the LOS; therefore, this study investigated whether different types of anaesthesia are associated with longer LOS. This study conducted a secondary analysis based on previously published data to investigate whether the two most commonly used types of anaesthesia were independently associated with LOS in patients undergoing unilateral TKA [7].

### Participants and methods

#### Study design

A retrospective cohort study was conducted to compare the LOS in patients who underwent unilateral TKA under the two most common types of anaesthesia. The objective independent variable was the type of anaesthesia, and the dependent variable was the LOS.

#### Study population

**Fig. 1:**
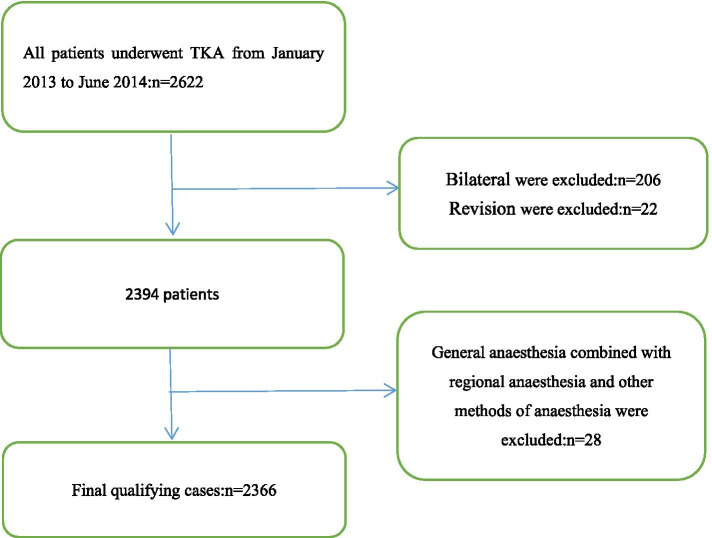
Flowchart of patient selection

This was a retrospective cohort study conducted at the Singapore General Hospital. All patients who underwent TKA from January 2013 to June 2014 (*n* = 2622). Patients who underwent bilateral TKA (*n* = 206) and those who underwent revision TKA (*n* = 22) were excluded. Patients who received GA combined with RA and other methods of anaesthesia were excluded (*n* = 28). The final number of qualifying cases was 2366. This study was approved by the Institutional Review Board prior to its initiation (SingHealth Centralized Institutional Review Board (CIRB) 2014/651/D) [7]. (Fig. 1) The informed consent of the participants was not required for this study because it was a retrospective cohort study. Written informed consent was waived by the SingHealth CIRB because our study did not involve the privacy or treatment of patients.

Abdullah et al. uploaded the raw data of their research to the Datadryad website (www.datadryad.org) and granted the Datadryad site ownership of the original data [7]. Therefore, we were able use these data for secondary analyses of different hypotheses without infringing on the rights of the authors.

#### Variables

We obtained the data of patients who underwent unilateral TKA, including the variables we needed, from the clinical information system of Singapore General Hospital. We considered the two types of anaesthesia as categorical variables, and unilateral TKA anaesthesia was divided into GA and RA.

We considered LOS a continuous variable, and then we generated the categorical variable based on a cut-off point of 6, defining an LOS longer than 6 days as an extended LOS. This cut-off was selected because it represents the >75th percentile LOS of the whole sample. The use of the 75th percentile to define prolonged LOS is consistent with other studies [7].

In this study, we obtained the following types of covariates from the database: (1) demographic data; (2) variables that can affect the type of anaesthesia or LOS; and (3) other diseases. The following variables were used to construct the fully adjusted model: (1) continuous variables: Age, BMI, Hb, LOS, duration of surgery; (2) categorical variables: Sex, ethnicity, American Society of Anesthesiologists (ASA) status, smoking, obstructive sleep apnea (OSA), diabetes mellitus (DM), ischemic heart disease (IHD), congestive cardiac failure (CCF), cerebrovascular accident (CVA), creatinine > 2 mg/dl, DM on insulin, day of week of operation.

The study protocol was performed in accordance with the relevant guidelines.

### Statistical analysis

The baseline characteristics of the participants are expressed as the mean ± standard deviation (Gaussian distribution) or median (range) (skewed distribution) for continuous variables and as percentages for categorical variables. We used χ2 (categorical variables), Student’s t test (normal distribution), or the Mann-Whitney U test (skewed distribution) to test for differences among the anaesthesia groups (bisected). The data analysis process of this study was based on three questions: (1) what is the relationship between anaesthesia and LOS?; (2) which factors modify or interfere with the relationship between anaesthesia and LOS?; and (3) after adjustment for interference factors or after the stratified analysis, what is the true relationship between anaesthesia and LOS? Therefore, data analysis can be summarized in three steps. Step 1: We used univariate and multivariate linear regression models to test the link between the two types of anaesthesia and LOS (days) with three distinct models, as presented in Table 3, and we used univariate and multivariate binary logistic regression models to test the connection between the two types of anaesthesia and LOS > 6 days with three distinct models, as presented in Table 4. The three models were a crude model, in which no covariates were adjusted; model 1, which was adjusted only for sociodemographic data (age, sex, ethnicity); model 2, which included the adjustments in model 1+ the other covariates presented in Table 1 [8]. Step 2: Subgroup analyses were performed using stratified linear regression models. For continuous variables, we first converted the variables to categorical variables according to the clinical cut-off point or tertile and then performed an interaction test. Tests for the effect modification of subgroup indicators were followed by the likelihood ratio test [9]. To ensure the robustness of the data analysis, we performed a sensitivity analysis. All analyses were performed with the statistical software packages R (http://www.R-project.org, The R Foundation) and EmpowerStats (http://www.empowerstats.com, X&Y Solutions, Inc., Boston, MA). *P* values less than 0.05 (two-sided) were considered statistically significant [10].

**Table 1:** Baseline Characteristics of participants underwent unilateral TKA (*N* = 2366)

Anaesthesia	Total	RA	GA	*P*-value
N	2366	1560	806	
Age (years),Mean ± SD	66.57 ± 8.23	67.46 ± 8.01	64.86 ± 8.39	< 0.001
BMI,Mean ± SD	27.68 ± 5.70	27.58 ± 6.16	27.87 ± 4.68	0.247
Hb(g/dL),Mean ± SD	13.08 ± 1.45	13.07 ± 1.42	13.09 ± 1.52	0.747
LOS,Mean (SD) Median (Q1-Q3)	5.37 (4.87) 4.00 (3.00–6.00)	5.09 (3.98) 4.00 (3.00–5.00)	5.92 (6.20) 4.00 (4.00–6.00)	< 0.001
Duration of surgery (mins), Mean ± SD	80.52 ± 22.81	79.93 ± 22.19	81.67 ± 23.95	0.079
Sex, n (%)				0.003
Female	1794 (75.82%)	1154 (73.97%)	640 (79.40%)	
Male	572 (24.18%)	406 (26.03%)	166 (20.60%)	
Ethnicity, n (%)				0.019
Chinese	1987 (83.98%)	1334 (85.51%)	653 (81.02%)	
Malay	169 (7.14%)	103 (6.60%)	66 (8.19%)	
Indian	137 (5.79%)	76 (4.87%)	61 (7.57%)	
Others	73 (3.09%)	47 (3.01%)	26 (3.23%)	
ASA Status				0.007
1	161 (6.80%)	91 (5.83%)	70 (8.68%)	
2	2058 (86.98%)	1381 (88.53%)	677 (84.00%)	
3	147 (6.21%)	88 (5.64%)	59 (7.32%)	
Smoking, n (%)	225 (9.51%)	161 (10.32%)	64 (7.94%)	0.061
OSA, n (%)	214 (9.04%)	144 (9.23%)	70 (8.68%)	0.661
DM, n (%)	446 (18.85%)	293 (18.78%)	153 (18.98%)	0.906
IHD, n (%)	127 (5.37%)	83 (5.32%)	44 (5.46%)	0.887
CCF, n (%)	18 (0.76%)	16 (1.03%)	2 (0.25%)	0.039
CVA, n (%)	45 (1.90%)	29 (1.86%)	16 (1.99%)	0.831
Creatinine > 2 mg/dl, n (%)	18 (0.76%)	11 (0.71%)	7 (0.87%)	0.657
DM on insulin, n (%)	39 (1.65%)	25 (1.60%)	14 (1.74%)	0.907
Day of week of operation, n (%)				0.153
Monday	390 (16.48%)	244 (15.64%)	146 (18.11%)	
Tuesday	539 (22.78%)	354 (22.69%)	185 (22.95%)	
Wednesday	405 (17.12%)	276 (17.69%)	129 (16.00%)	
Thursday	533 (22.53%)	368 (23.59%)	165 (20.47%)	
Friday	378 (15.98%)	247 (15.83%)	131 (16.25%)	
Saturday	121 (5.11%)	71 (4.55%)	50 (6.20%)	

*Abbreviations*: *GA* General anaesthesia, *RA* Regional anaesthesia, *LOS* Length of stay, *ASA* American Society of Anesthesiologist Physical Status, *OSA* Obstructive sleep apnea, *DM* Diabetes mellitus, *IHD* Ischaemic heart disease, *CCF* Congestive cardiac failure, *CVA* Cerebrovascular accidents, *TKA* Total knee arthroplasty

## Results

### Baseline characteristics of selected participants

A total of 2366 participants were selected for the final data analysis after screening with the inclusion and exclusion criteria (see Fig. 1 for a flow chart). We show the baseline characteristics of the selected participants in Table 1 according to anaesthesia type. In general, the average age of the 2366 selected participants was 66.57 ± 8.23 years, and approximately 24.18% were male. There were 1560 patients in the RA group and 806 cases in the GA group. No statistically significant differences were detected in BMI, Hb, Duration of surgery,smoking, OSA, DM, IHD, CVA, creatinine > 2 mg/dl, DM on insulin, or day of the week of operation between the different anaesthesia groups (all *p* values > 0.05). LOS were higher in the GA group than in the RA group (*P* < 0.05). The mean age of the GA group (64.86 ± 8.39) was lower than that of the RA group (67.46 ± 8.01) (*P* < 0.05). The proportion of male patients was 20.60% in the GA group and 26.03% in the RA group (*P* < 0.05). Additionally, there were differences in ethnicity, ASA status and CCF between the two groups (*P* < 0.05).

### Univariate analysis

The results of the univariate analyses are presented in Table 2. The univariate linear regression showed that male sex; Malay, Indian and other ethnicity; BMI group; ASA 2; duration of surgery (min) between 60 and 120 min; smoking; OSA; CVA; DM on insulin; and surgery performed on a Tuesday, Wednesday, or Friday (all of which vs ref) were not associated with LOS. We also found that surgery performed on a Thursday (− 1.29, − 1.92 --0.66 vs ref) or Saturday (− 1.31, − 2.30--0.32 vs ref) were negatively associated with LOS. In contrast, univariate analysis showed that age 70–80 years (1.14,0 .58–1.70 vs ref), age ≥ 80 years (2.43, 1.52–3.35 vs ref), ASA 3 (3.98, 2.91–5.05 vs ref), Hb group ≥11 to 13 (0.25, 1.07–0.66 vs ref), Hb group < 11 (1.31,2.88–2.09 vs ref), duration of surgery ≥120 min (1.74, 0.78–2.69 vs ref), DM (0.94, 0.44–1.44 vs ref), IHD (1.71, 0.85–2.58 vs ref), CCF (5.90, 3.65–8.14 vs ref), and creatinine > 2 mg/dl (6.53, 4.28–8.77 vs ref) were positively correlated with LOS. Thus, the univariate analysis showed that age ≥ 70 years, ASA 3, Hb < 13 g/dL, operation duration ≥120 min, DM, IHD, CCF, creatinine > 2 mg/dL, and other factors are associated with greater LOS.

**Table 2:** Univariate analysis for LOS (Days)

	Statistics	LOS (Days) β(95%CI) *P-*value
Age (years) group		
< 60	472 (19.95%)	Reference
> =60, < 70	1011 (42.73%)	0.30 (− 0.22, 0.83) 0.2604
> =70, < 80	744 (31.45%)	1.14 (0.58, 1.70) < 0.0001
> =80	139 (5.87%)	2.43 (1.52, 3.35) < 0.0001
Sex		
Female	1794 (75.82%)	Reference
Male	572 (24.18%)	−0.14 (− 0.60, 0.32) 0.5539
Ethnicity		
Chinese	1987 (83.98%)	Reference
Malay	169 (7.14%)	−0.48 (−1.24, 0.28) 0.2185
Indian	137 (5.79%)	0.00 (− 0.84, 0.84) 0.9975
Others	73 (3.09%)	0.63 (−0.51, 1.77) 0.2790
BMI group		
< 25	726 (31.44%)	Reference
> =25, < 30	963 (41.71%)	−0.28 (−0.73, 0.16) 0.2159
> =30, < 35	467 (20.23%)	−0.38 (− 0.92, 0.16) 0.1692
> =35	153 (6.63%)	0.30 (−0.51, 1.11) 0.4650
ASA Status		
1	161 (6.80%)	Reference
2	2058 (86.98%)	0.45 (− 0.32, 1.22) 0.2526
3	147 (6.21%)	3.98 (2.91, 5.05) < 0.0001
Hb(g/dL) group		
> =13	1314 (55.54%)	Reference
> =11, < 13	888 (37.53%)	0.66 (0.25, 1.07) 0.0018
< 11	164 (6.93%)	2.09 (1.31, 2.88) < 0.0001
Duration of surgery (mins) group		
< 60	320 (13.52%)	Reference
> =60, < 120	1903 (80.43%)	0.26 (−0.31, 0.84) 0.3678
> =120	143 (6.04%)	1.74 (0.78, 2.69) 0.0004
Smoking	225 (9.51%)	−0.49 (−1.16, 0.18) 0.1525
OSA	214 (9.04%)	−0.62 (− 1.30, 0.07) 0.0765
DM	446 (18.85%)	0.94 (0.44, 1.44) 0.0002
IHD	127 (5.37%)	1.71 (0.85, 2.58) 0.0001
CCF	18 (0.76%)	5.90 (3.65, 8.14) < 0.0001
CVA	45 (1.90%)	1.21 (−0.23, 2.64) 0.0993
Creatinine > 2 mg/dl	18 (0.76%)	6.53 (4.28, 8.77) < 0.0001
DM on insulin	39 (1.65%)	0.30 (−1.25, 1.84) 0.7078
Day of week of operation		
Monday	390 (16.48%)	Reference
Tuesday	539 (22.78%)	−0.31 (− 0.94, 0.32) 0.3404
Wednesday	405 (17.12%)	−0.62 (−1.30, 0.05) 0.0691
Thursday	533 (22.53%)	−1.29 (− 1.92, − 0.66) < 0.0001
Friday	378 (15.98%)	0.27 (−0.41, 0.96) 0.4340
Saturday	121 (5.11%)	−1.31 (−2.30, −0.32) 0.0095

*Abbreviations*: *LOS* Length of stay, *ASA* American Society of Anesthesiologist Physical Status, *OSA* Obstructive sleep apnea, *DM* Diabetes mellitus, *IHD* Ischaemic heart disease, *CCF* Congestive cardiac failure, *CVA* Cerebrovascular accidents

### Unadjusted and adjusted linear and logistic regression results

In this study, we constructed three models to analyse the independent effects of two types of anaesthesia on LOS (univariate and multivariate linear and logistic regression). The effect sizes (β and OR (odds ratio), β for LOS, OR for LOS > 6 days) and 95% confidence intervals are listed in Tables 3 and 4. In the unadjusted (crude) model, the effect size of 0.84 for LOS in the unadjusted model indicated that the LOS was higher in the GA group than in the RA group (0.84, 95% CI (0.43, 1.25)), and the difference was statistically significant (*P* < 0.05). In the minimally adjusted model (model 1), the LOS was higher in the GA group than in the RA group (1.08, 95% CI (0.67, 1.49)), and the difference was statistically significant (*P* < 0.05). In the fully adjusted model (model 2) (adjusted for all covariates presented in Table 1), LOS was higher in the GA group than in the RA group (0.93, 95% CI (0.54, 1.32)), and the difference was statistically significant (*P* < 0.05). In the unadjusted (crude) model, the effect size of 1.33 for LOS > 6 days in the unadjusted model indicated that the proportion of patients with an LOS > 6 days was 33% higher in the GA group than in the RA group (1.33, 95% CI (1.08, 1.64)), and the difference was statistically significant (*P* < 0.05). In the minimally adjusted model (model 1), the proportion of patients with an LOS > 6 days was 51% higher in the GA group than in the RA group (1.51, 95% CI (1.21, 1.87)), and the difference was statistically significant (*P* < 0.05). In the fully adjusted model (model 2) (adjusted all covariates presented in Table 1), the proportion of patients with an LOS > 6 days was 45% higher in the GA group than in the RA group (1.45, 95% CI (1.15, 1.84)), and the difference was statistically significant (*P* < 0.05).

**Table 3:** Relationship between Two types of anaesthesia and LOS (Days)

Outcome	LOS (Days) β(95%CI) *P-*value		
Anaesthesia	Crude Model	Model I	Model II
RA	Reference	Reference	Reference
GA	0.84 (0.43, 1.25) < 0.0001	1.08 (0.67, 1.49) < 0.0001	0.93 (0.54, 1.32) < 0.0001

Model I adjusted for age, sex, ethnicity

Model II adjusted for age, sex, ethnicity, BMI,ASA Status, Hb, duration of surgery, smoking, OSA, DM, IHD, CCF, CVA, Creatinine > 2 mg/dl, DM on insulin, Day of week of operation

*Abbreviations*: *LOS* Length of stay, *GA* General anaesthesia, *RA* Regional anaesthesia, *OR* Odds ratio, *CI* Confidence interval

**Table 4:** Relationship between Two types of anaesthesia and LOS > 6 Days

Outcome	LOS > 6 Days OR(95%CI) *P-*value		
Anaesthesia	Crude Model	Model I	Model II
RA	Reference	Reference	Reference
GA	1.33 (1.08, 1.64) 0.0084	1.51 (1.21, 1.87) 0.0003	1.45 (1.15, 1.84) 0.0016

Model I adjusted for age, sex, ethnicity

Model II adjusted for age, sex, ethnicity, BMI,ASA Status, Hb, duration of surgery, smoking, OSA, DM, IHD, CCF, CVA, Creatinine > 2 mg/dl, DM on insulin, Day of week of operation

*Abbreviations*: *LOS* Length of stay, *GA* General anaesthesia, *RA* Regional anaesthesia, *OR* Odds ratio, *CI* Confidence interval

### Subgroup analysis

We used age group, sex, ethnicity, BMI group, ASA status, HB group, duration of surgery group, smoking, OSA, DM, IHD, CCF, CVA, creatinine > 2 mg/dl, DM on insulin, Day of week of operation as the stratification variables to observe the trend of effect sizes in these variables (Table 5). We observed only a small number of interactions, including those between age group, BMI group, IHD, CCF, and creatinine > 2 mg/dl, based on our a priori specification (all *P* values for interaction < 0.05).

**Table 5:** Effect size of two types of anaesthesia on LOS (Days) in prespecified and exploratory subgroups in Each Subgroup

Anaesthesia	LOS (Days)			
	N	β	95%CI	Interaction *P*-value
Age (years) group				0.0072
< 60	472	0.45	(− 0.03, 0.92)	
> =60, < 70	1011	0.56	(− 0.07, 1.20)	
> =70, < 80	744	1.99	(1.15, 2.83)	
> =80	139	2.46	(−0.29, 5.20)	
Sex				0.0588
Female	1794	0.62	(0.19, 1.05)	
Male	572	1.58	(0.49, 2.67)	
Ethnicity				0.8872
Chinese	1987	0.86	(0.38, 1.34)	
Malay	169	0.78	(−0.04, 1.60)	
Indian	137	0.40	(− 0.53, 1.32)	
Others	73	1.53	(−0.46, 3.52)	
BMI group				0.0067
< 25	726	1.87	(1.06, 2.67)	
> =25, < 30	963	0.28	(−0.36, 0.93)	
> =30, < 35	467	0.38	(−0.26, 1.01)	
> =35	153	0.96	(−0.17, 2.09)	
ASA Status				0.5077
1	161	−0.01	(− 0.74, 0.73)	
2	2058	0.84	(0.46, 1.21)	
3	147	1.16	(0.46, 1.21)	
Hb(g/dL) group				0.9170
< 11	164	1.08	(−1.04, 3.19)	
> =11, < 13	888	0.92	(0.17, 1.67)	
> =13	1314	0.79	(0.32, 1.26)	
duration of surgery (mins) group				0.5324
< 60	320	1.00	(0.28, 1.72)	
> =60, < 120	1903	0.72	(0.25, 1.18)	
> =120	143	1.63	(−0.77, 4.03)	
Smoking				0.1232
No	2141	0.93	(0.48, 1.37)	
Yes	225	−0.23	(−1.13, 0.68)	
OSA				0.5106
No	2152	0.88	(0.43, 1.32)	
Yes	214	0.39	(−0.40, 1.18)	
DM				0.2677
No	1920	0.72	(0.30, 1.15)	
Yes	446	1.32	(0.12, 2.51)	
IHD				0.0002
No	2239	0.65	(0.25, 1.05)	
Yes	127	4.05	(1.07, 7.02)	
CCF				0.0423
No	2348	0.91	(0.52, 1.29)	
Yes	18	−5.88	(−36.03, 24.28)	
CVA				0.3150
No	2321	0.81	(0.39, 1.23)	
Yes	45	2.34	(0.34, 4.33)	
Creatinine > 2 mg/dl				< 0.0001
No	2090	1.06	(0.64, 1.47)	
Not recorded	258	−0.43	(−1.55, 0.68)	
Yes	18	−7.68	(−26.62, 11.27)	
DM on insulin				0.1413
No	1764	1.07	(0.57, 1.57)	
Not recorded	563	0.09	(−0.62, 0.79)	
Yes	39	1.04	(−1.10, 3.17)	
Day of week of operation				0.1282
Monday	390	1.08	(−0.34, 2.49)	
Tuesday	539	0.37	(−0.29, 1.04)	
Wednesday	405	0.40	(−0.28, 1.08)	
Thursday	533	0.60	(0.01, 1.18)	
Friday	378	2.08	(0.60, 3.57)	
Saturday	121	0.15	(−0.69, 0.99)	

*Abbreviations*: *LOS* Length of stay, *ASA* American Society of Anesthesiologist Physical Status, *OSA* Obstructive sleep apnea, *DM* Diabetes mellitus, *IHD* Ischaemic heart disease, *CCF* Congestive cardiac failure, *CVA* Cerebrovascular accidents

## Discussion

The present study was a retrospective cohort study comparing the effects of GA and RA on LOS in patients undergoing unilateral TKA. Our study showed that unilateral TKA performed under GA lasted 0.93 days longer than that performed under RA after adjusting for potential risk factors associated with LOS. Patients who received GA had a 45% higher rate of LOS > 6 days than those who received RA. Confounding factors including age, BMI, Hb, duration of surgery, sex, ethnicity, ASA, smoking, OSA, DM, DM on insulin, IHD, CCF, CVA, creatinine > 2 mg/dl, and day of week of operation may affect the LOS [7]. To confirm this, we used subgroup analysis and found that only age, BMI, IHD, CCF, and Creatinine > 2 mg/dl could influence the relationship between types of anaesthesia and LOS. After adjusting these covariables above, the direction of types of anaesthesia and LOS in the three models was consistent, and the results were stable and reliable.

Due to the continuous improvement of the global social economy and medical and health conditions and to the ageing of the population, the incidence of osteoarthritis is increasing yearly, affecting the daily work and life of middle-aged and elderly people. The knee joint is the most common location of osteoarthritis in this population, mainly due to the degeneration of knee articular cartilage. The main symptoms are local pain in the knee joint, local joint deformity and changes in mobility, which also lead to anxiety, poor sleep and discomfort. At present, arthroplasty is the most important treatment for chronic osteoarthritis of the knee [11]. The adverse effects and discomfort caused by cartilage degeneration in the knee joint can be relieved by arthroplasty.

Shortening the LOS for TKA patients can increase the hospital bed turnover rate and reduce patients’ wait time for elective surgery. Additionally, it can reduce the cost of treatment, which reduces the burden on medical insurance, reduces patients’ medical expenses, and saves medical and health resources [12]. Furthermore, reducing the LOS can reduce the probability of cross-infection among patients [13] and shorten the time of direct contact between patients and doctors, which, especially during epidemics, can reduce the potential risk of disease transmission. Yehoshua Gleicher et al. suggested that (1) perioperative peripheral nerve block, (2) prophylactic antiemetic drugs, (3) avoidance of routine preoperative catheterization, and (4) preoperative patient education can effectively shorten the LOS [14]. Shortening the LOS can also reduce the incidence of deep vein thrombosis [15], reduce the perioperative blood transfusion rate [16], motivate patients to perform early functional exercises, and reduce the likelihood of fracture and infection around the prosthesis, enabling the body to adapt to the placement of the prosthesis earlier.

Chapman Wei et al. analysed the data from the American College of Surgeons’ NSQIP database and concluded that in patients undergoing revision total knee replacement, RA has a lower incidence of postoperative complications, a lower probability of prolonged LOS and a lower incidence of perioperative blood transfusion than GA [17]. Anahi Perlas et al. suggested that RA during total hip and knee arthroplasty was associated with a lower 30-day postoperative mortality and a shorter LOS than GA [18]. Eva E. Morwald et al. suggested that obstructive sleep apnoea was associated with a higher incidence of perioperative complications and that reducing the use of opioids could reduce the incidence of perioperative complications [19]. Gulraj S. Matharu suggested that RA was associated with reduced LOS, readmission, and complications after THA and TKA surgery and suggested that RA should be used as a reference standard for patients undergoing THA and TKA surgery [20]. The Ipek S. Edipoglu study showed that TKA patients who received RA showed lower cortisol, higher insulin, and lower glucose levels, all of which could avoid the early occurrence of POCD after surgery. POCD is a serious complication associated with total knee arthroplasty (TKA) and has been shown to increase LOS, cause functional impairment, and morbidity [21]. Their conclusions are consistent with our findings. However, the results of some other studies are inconsistent with our findings. Adam Hart et al. analysed patients in the United States and Canada undergoing total hip arthroplasty and TKA. In the United States, there was a high proportion of GA use. In Canada, RA was much more preferred in TKA than GA. Patients undergoing the procedures in US hospitals also had substantially shorter LOS. This conclusion indicates that more effective postoperative nursing and discharge planning can reduce the LOS [22]. Riku Palanne et al. found that spinal anaesthesia was associated with a higher incidence of postoperative pain and vomiting than GA, and there was no difference in LOS [4]. According to the study of Yining Lu et al., spinal anaesthesia was associated with reduced perioperative adverse events and reduced operating room time, and there was no difference in the LOS under different anaesthesia methods [23]. We analysed these studies with results that were inconsistent with ours, and we speculated that the differences might be due to the following factors: (1) the study population was different; (2) the relationship between anaesthesia method and LOS was not analysed separately; and (3) the conditions and methods of postoperative rehabilitation and nursing differ in different countries and regions.

Our study has some strengths. (1) The relationship between the two types of anaesthesia and LOS in unilateral TKA was analysed independently. (2) There were few missing data and reliable and stable results. (3) This study was an observational study, so it was susceptible to potential confounders; however, we used strict statistical adjustment to minimize residual confounders. (4) We treated the target independent variables as continuous and categorical variables, which can reduce the contingency in data analysis and enhance the robustness of the results. (5) For different subgroups of this study, relatively stable conclusions were drawn.

Limitations of this study include the following: (1) The subjects were patients undergoing unilateral TKA; therefore, the results are not applicable to patients undergoing other types of surgery,; (2) Because of the small number of cases, we excluded patients who underwent GA combined with RA and other anaesthesia methods, and the findings of this study cannot be used to make conjectures regarding that population.

There are still some shortcomings in this paper. For example, this paper was a retrospective study, and the data used were collected from the clinical data of a single centre; therefore, there may be differences in treatment strategies, ethnicities, etc. More data from other centres are needed to fine-tune the model’s predictive performance. There may be some influencing factors that were not included in the paper, including subjective factors such as patient income, medical expenses and medical insurance, and potential factors that were not included may have also had some impact on the results.

Due to current medical factors, such as medical insurance cost control efforts, large numbers of waitlisted inpatients, and reduced numbers of specialized orthopaedic beds at the hospital, patient LOS has certain constraints, and the entire process of knee rehabilitation cannot be completed during hospitalization. Under the existing conditions, hospitals can only provide early postoperative rehabilitation training, and only the angle of knee flexion and straightening, local pain, and inspection are addressed during hospitalization. Choosing the right anaesthesia method to reduce the patient’s hospitalization duration is a very effective method for shortening the LOS, and the clinical pathway for TKA fast rehabilitation surgery needs ongoing improvement and development [24].

## Conclusion

Our study showed that GA is associated with a longer LOS during unilateral TKA compared with RA. This can be taken into account when choosing the type of anaesthesia before surgery. This finding needs to be validated in future studies.

## Data Availability

Data can be down-loaded from“DATADRYAD”database. Dryad data package: Abdullah HR, Sim E, Hao Y, Lin G, Liew GHC, Lamoureux EL,Tan MH (2017). Data from: Association between preoper-ative anemia with length of stay among patients undergoing primary total knee arthroplasty in Singapore:a single-center retrospective study. Dryad Digital Reposi-tory. 10.5061/dryad.73250.
